# Satisfaction with Care in Late Stage Parkinson's Disease

**DOI:** 10.1155/2019/2593547

**Published:** 2019-06-09

**Authors:** Kristina Rosqvist, Peter Hagell, Susanne Iwarsson, Maria H Nilsson, Per Odin

**Affiliations:** ^1^Lund University, Faculty of Medicine, Department of Clinical Sciences Lund, Neurology, Lund, Sweden; ^2^The PRO-CARE Group, Faculty of Health and Science, Kristianstad University, Kristianstad, Sweden; ^3^Lund University, Faculty of Medicine, Department of Health Sciences, Lund, Sweden; ^4^Memory Clinic, Skåne University Hospital, Malmö, Sweden; ^5^Department of Neurology, Skåne University Hospital, Lund, Sweden

## Abstract

In late stage Parkinson's disease (PD) (i.e., Hoehn and Yahr (HY) stages IV-V), both motor and nonmotor symptoms (NMS) are pronounced, and the patients become increasingly dependent on help in their daily life. Consequently, there is an increasing demand on health-care and social care resources for these patients and support for their informal caregivers. The aim of this study was to assess satisfaction with care in late stage PD patients and to identify factors associated with satisfaction with care. Moreover, to assess their informal caregivers' satisfaction with support and to identify factors associated with caregivers' satisfaction with support. Factors potentially associated with satisfaction with care/support were assessed in 107 late stage PD patients and their informal caregivers (*n*=76) and entered into multivariable logistic regression analyses. Fifty-eight (59%) of the patients and 45 (59%) of the informal caregivers reported satisfaction with their overall care/support. Patients satisfied with their care reported higher independence in activities of daily living (ADL) (Katz ADL index; *P*=0.044), less depressive symptoms (Geriatric Depression Scale, GDS-30; *P*=0.005), and higher individual quality of life (QoL) (Schedule for the Evaluation of Individual Quality of Life Questionnaire, SEIQoL-Q; *P*=0.036). Multivariable logistic regression analyses identified depressive symptoms (*P*=0.015) and independence in ADL (*P*=0.025) as independently associated with satisfaction with care. For informal caregivers, the analyses identified patients' HY stage (*P*=0.005) and caregivers' QoL (Alzheimer's Carers Quality of Life Inventory, ACQLI; *P*=0.012) as independently associated with satisfaction with caregiver support. The results indicate that an effective both pharmacological and nonpharmacological PD therapy is important, to adequately treat motor and NMS (e.g., depressive symptoms) in order to improve depressive symptoms and patient independence in ADL. This may benefit not only the patients, but also their informal caregivers.

## 1. Introduction

In the late and most severe stage of Parkinson's disease (PD), i.e., Hoehn and Yahr (HY) stages IV-V [1], both motor and nonmotor symptoms (NMS) are pronounced [2–4], and the patients become increasingly dependent on help from others in their daily life [1, 5].

Late stage PD has been estimated to constitute about 15–20% of the PD population [6], which prevalence is expected to increase due to increased longevity and improved health care [5, 7, 8]. Moreover, as PD is a progressive disease, the burden on informal/family caregivers is likely to increase during the late stage of the disease [9]. Consequently, there is an increasing demand on health-care and social care resources for these patients and support for their informal/family caregivers [10].

Previous research has shown that PD causes a great burden for the patients' family caregivers, which affects their own physical health, relationships, and mood [11]. It is essential to recognize and manage caregiver burden, for the well being of both the patient and the family caregiver, as failure to do so may lead to exhaustion for the caregiver and premature institutionalization for the patient [9].

There is a growing focus on patient-centered health care, which includes taking into consideration experiences of patients and their informal caregivers when it comes to satisfaction with the care and support they receive [12]. However, there is limited knowledge on which factors are associated with satisfaction with care in PD in general [13] and especially in late stage PD. An increased knowledge regarding factors associated with satisfaction with care and support in late stage PD could serve as an important base for optimizing health and social care for this patient group.

The aim of this study was to describe and assess satisfaction with care in a sample of patients with late stage PD in Sweden and to identify factors associated with patient satisfaction with care as well as to describe and assess their informal caregivers' satisfaction with support and to identify factors associated with caregivers' satisfaction with support.

## 2. Material and Methods

### 2.1. Participants and Recruitment

Participants were recruited from the southern region of Sweden through neurology departments and the municipality-based health-care system. A total of 166 individuals were identified for the study and were contacted by post (through their treating physicians) and then by telephone. In most cases, the contact was made through an informal caregiver or nursing home/health-care personnel. Out of these, 53 declined, and six were noneligible.

Inclusion criteria were HY stages IV and V in “on” and/or having a substantial need of help with ADL (defined here as ≤ 50% on the Schwab and England Scale) [14], as well as having been diagnosed with idiopathic PD for a minimum of seven years. Exclusion criteria were cognitive symptoms that started before the PD diagnosis as well as symptomatic Parkinsonism (such as drug-induced Parkinsonism or normal pressure hydrocephalus). The present study was a Swedish substudy of the European multicenter project Care for Late Stage Parkinsonism (CLaSP) [15], where additional assessments were carried out at the Lund center, Sweden.

The study was approved by the Regional Ethical Review Board in Lund, Sweden (JPND HC-559-002). Written informed consent was obtained by the participants.

### 2.2. Procedure and Clinical Evaluation

Clinical assessments were made, and questionnaires were answered by patients and their informal family caregiver, if such a person existed and was available, during home visits. A number of self-administered rating scales for patients and informal caregivers were sent by post beforehand as well as returned by post afterwards. Any questions regarding the questionnaires were answered by the person making the assessments at the time of the home visit. All assessments were carried out during a time of the day when the patient normally was doing his/her best.

Independence in activities of daily living (ADL) was assessed by the Katz ADL index [16] (score range 0–6, 6 = independence, higher = better). Motor function was assessed by the motor section of the Unified PD Rating Scale (UPDRS; part III, score range 0–108, higher = worse) [17]. Nonmotor symptomatology was assessed by the NMS Scale (NMSS; score range 0–360, higher = worse) [18]. Cognitive function was assessed by the Mini-Mental State Examination (MMSE, score range 0–30, higher = better) [19], where a score ≥ 24 was regarded as normal [20]. Depressive symptoms were assessed with the Geriatric Depression Scale (GDS-30; score range 0–30, higher = worse) [21]. Cut-off for screening of depression (scores ≥10) has been validated for PD [22]. Generic health status was assessed by the EuroQol 5 Dimensions (EQ-5D-3 L; score range 0-1, higher = better) and visual analogue scale (score range 0–100, higher = better) [23, 24]. Individual QoL was assessed by the Schedule for the Evaluation of Individual QoL Questionnaire (SEIQoL-Q; score range 0–100, higher = better), which was translated from German to Swedish through validated forward-back translation [25]. Self-rated health status was assessed by the Nottingham Health Profile (NHP), which consists of six sections: emotional reactions, sleep, energy, pain, physical mobility, and social isolation (each section score range 0–100, higher = worse) [26–28]. Each section score was for the simple and multivariable logistic regression analyses dichotomized as better (0 = 0–50) and worse (1 = 51–100). Life satisfaction was assessed by the Life Satisfaction Questionnaire (LiSat-11; each item score range 1–6, higher = better) [29]; for the simple and multivariable logistic regression analyses, the item responses were dichotomized as not satisfied (0 = from very dissatisfied to rather satisfied; i.e., scores 1–4) and satisfied (1 = satisfied and very satisfied; i.e., scores 5-6). This dichotomization has previously been used by the developers of the instrument [29] as well as in previous PD research [30, 31]. Patient satisfaction with overall care and caregiver satisfaction with overall support were assessed by CLaSP [15] study-specific questions with five response categories: very satisfied, satisfied, neutral, unsatisfied, and very unsatisfied. The responses were dichotomized as satisfied (1 = very satisfied or satisfied) and not satisfied (0 = neutral, unsatisfied, or very unsatisfied) and used as the dependent variable. Caregiver QoL was assessed with Alzheimer's Carers' QoL Inventory (ACQLI; score range 0–30, higher = worse), which has been found useful also among caregivers in PD [32].

Complimentary information on whether the patient had been in contact with movement disorder specialist (MDS), PD-specialized nurse, or physiotherapist/occupational therapist/speech and language therapist during the past year was retrieved from the medical records. Data on whether the patients had professional home health care were collected from a study-specific questionnaire.

### 2.3. Statistical Analyses

Descriptive and clinical data are given by median and first and third quartiles (q1–q3) and frequencies and percentages, as appropriate. Differences were tested statistically with simple logistic regression analyses. *P* values of <0.05 were considered significant. For the patients, ten independent variables with *P* values <0.2 from the simple logistic regression analyses and two variables with *P* values >0.2 that were considered clinically relevant were simultaneously entered into a multivariable logistic regression model. Two independent variables with *P* < 0.2 were excluded: SEIQoL due to a high number of missing and EQ-5D VAS due to a high number of independent variables in relation to the number of participants, in order to keep the number of independent variables entered into the multivariable model limited. For informal caregivers, nine independent variables were entered into a multivariable logistic regression model. *P* values were inspected, and the variable with the highest *P* value was manually removed from the model through stepwise backward method, which was repeated until the remaining independent variables in the model had *P* values <0.1. All analyses were performed using IBM SPSS version 24.0 (IBM 211 Corporation, Armonk, NY, USA).

## 3. Results

### 3.1. Demographic Data and Clinical Background Assessments

A total of 107 patients participated in this study, 62 (58%) of them were men. The median (q1–q3) age was 78 (73–84) years. Seventy-nine (74%) were in HY stage IV and 28 (26%) in stage V. Sixty-seven (62%) lived in ordinary housing and 40 (37%) in nursing home. The majority (*n*=89; 83%) received professional home health care (Table 1).

The median (q1–q3) UPDRS III score was 40 (29–53). The median (q1–q3) NMSS score was 91 (55–128). The median (q1–q3) MMSE score was 22 (18–27). The median (q1–q3) GDS-30 score was 11 (8–16). Sixty-two (58%) of the participants reported depressive symptoms that were above the cut-off for screening of depression [22].

### 3.2. Satisfaction with Care and Informal Caregiver Support

Of the 99 (93%) individuals who had answered the question, 58 (59%) were satisfied with their care. Five of the eight participants who had not responded to this question had severe difficulties in participating and presented a more severe disease (50% were in HY V) with severe motor and nonmotor symptomatology and markedly worse cognitive impairment.

Regarding the patients' informal caregivers, 45 (59%) of the 76 individuals who had responded to the question were satisfied with their support. Out of them, 57 (75%) were the spouse/partner, 18 (24%) were a son/daughter, and one was a sibling to the patient. In 27 (36%) cases out of the 76 replies from informal caregivers, both the patient and the informal caregiver had replied that they were satisfied with their care/support.

Comparisons between patients who were satisfied and patients who were not satisfied with their overall care indicated that those with higher satisfaction with care were more independent in ADL (*P*=0.044), showed less depressive symptoms (*P*=0.005), and rated their individual QoL better on the SEIQoL-Q (*P*=0.036). For more detailed information, see Table 2.

Comparisons between caregivers who were satisfied and caregivers who were not satisfied with their overall support identified the following variables as statistically significant: patient age (*P*=0.009), patient HY stage (*P*=0.004), patient motor function (UPDRS III) (*P*=0.015), and caregiver QoL (ACQLI) (*P*=0.036) (Table 3).

### 3.3. Multivariable Logistic Regression Analyses

The analyses identified depressive symptoms (*P*=0.015), independence in ADL (*P*=0.025), and satisfaction with somatic health (*P*=0.074; i.e., not statistically significant) as independently associated with satisfaction with care. That is, depressive symptoms were negatively associated with satisfaction with care and independence in ADL, and satisfaction with somatic health were positively associated with satisfaction with care (Table 4). In a further step, age and gender were added to the final model. However, the final results remained identical, and age and gender were highly nonsignificant (age *P*=0.912; gender *P*=0.766).

A separate multivariable regression model was performed for informal caregiver data. The analyses identified patient HY stage (*P*=0.005) and caregiver QoL (*P*=0.012) as independently associated with satisfaction with caregiver support, where patient HY stage was positively associated with satisfaction with caregiver support (i.e., informal caregivers reported higher satisfaction with support when the patient was in HY stage V compared to stage IV) and caregiver QoL was negatively associated with satisfaction with caregiver support (i.e., informal caregivers reported lower satisfaction with support when rating lower QoL) (Table 5). When analyzing different clinical (patient) variables in relation to caregiver satisfaction, for patients who lived at home, no other differences were found between caregivers satisfied with their support and those who were not (Table 6).

## 4. Discussion

In this Swedish sample of patients in late stage PD, 59% reported satisfaction with their overall care and 59% of their informal caregivers reported satisfaction with their overall caregiver support. Depressive symptoms, independence in ADL, and satisfaction with somatic health were identified as factors associated with whether the patient was satisfied with his or her overall care. Patients' HY stage and informal caregivers' QoL were associated with informal caregivers' satisfaction with support.

Surprisingly, none of the background variables such as age, gender, dwelling place, HY stage, or whether having a partner differed between the patients who were satisfied with their overall care and those who were not. This suggests that other factors may be more important for patients' satisfaction with their overall care, such as the presence of depressive symptoms, independence in ADL, and satisfaction with somatic health. These findings are in line with the results of another PD study, where satisfaction with care did not differ based on demographic variables such as age, gender, education, or disease duration [13]. Similarly to our finding that 59% of the patients reported satisfaction with their overall care, the previous study found that 57% of their participants expressed satisfaction with their care [13].

Fatigue (NHP-energy) is a variable that was close to significant in the univariate analyses and that seemed to matter, coming close to significant also in the multivariable model on satisfaction with care. Another variable coming close to being presented in the multivariable model was satisfaction with life as a whole. These factors may also matter for patients' satisfaction with care.

### 4.1. Depressive Symptoms

The results showed that depressive symptoms are one of the major factors negatively associated with satisfaction with care. Depressive symptoms have previously been identified as negatively associated with life satisfaction in late stage PD and as the strongest factor negatively associated with life satisfaction in all stages of PD [31]. As depressive symptoms are common in late stage PD [3, 5, 33, 34], it is important to identify and optimize treatment of depressive symptoms, not only to alleviate the symptoms per se, but also to enhance functional ability [35] and QoL [11, 36]. This may in turn lead to improved satisfaction with care.

Depressive symptoms in PD can be treated by antidepressive pharmacological treatment, i.e., serotonin-norepinephrine reuptake inhibitors (SNRIs), tricyclic antidepressants (TCAs), and probably serotonin selective reuptake inhibitors (SSRIs) as well as through nonpharmacological interventions such as cognitive behavioural therapy [37] and exercise [38]. Optimizing dopaminergic treatment is also essential, as there is a dopaminergic response on NMS in late stage PD, with pronounced effects particularly on mood [3]. For those with nonmotor fluctuations or depressive symptoms occurring only in motor “off” state, this may be the preferred approach [39, 40].

### 4.2. Independence in ADL

In late stage PD, patients have pronounced motor and NMS and become increasingly dependent on help from others in their daily life, both when it comes to transfers and ADL [1, 5]. It is previously known that NMS such as depressive symptoms and cognitive impairment contribute to disability and worsened ADL function [35]. Independence in ADL may be improved and function maintained for longer through appropriate physiotherapy and occupational therapy training [41]. In order to reach increased independence in ADL, multiple actions are likely needed from both pharmacological and nonpharmacological approaches when it comes to therapy for both motor and NMS. This could mean that good availability of health care specialized on this patient group is needed. However, this needs to be further demonstrated in intervention studies.

The evidence for the benefit of PD team and PD team rehabilitation is not very strong generally. However, clinical experience supports the value of these, which is reflected in the consensus recommendations within the National Swedish Guidelines for PD [42]. Further, previous research has suggested higher overall satisfaction with care when patients were treated by PD specialists compared to general neurologists [12, 13].

### 4.3. Informal Caregivers

For the patients' informal caregivers, the main results identified an association between patients' HY stage and informal caregivers' satisfaction with overall support, where the informal caregivers were more satisfied with their support when the patient was in the most severe stage of PD, i.e., HY stage V, as compared to stage IV. Moreover, for patients living at home, the informal caregivers still reported higher satisfaction with support when the patient was in HY stage V (Tables 5 and 6). An explanation to this could be a higher strain on informal caregivers when the patient is in stage IV compared to stage V, as all patients in stage V had home health care compared to 77% in stage IV. A second explanation could be that 61% of the patients in stage V lived in a nursing home, compared to 29% in stage IV. However, this is unlikely to be the main explanation, since the result was similar when analyzing the results of the participants living at home (Table 6). A third possible reason could be that patients in stage V are less mobile than those in stage IV, which in certain aspects could mean less strain on the informal caregiver, as patients in HY stage V are restricted to wheelchair or bed unless aided by another person [1], while in stage IV, the patient walks independently in spite of a severely disabling disease, which could mean severe balance impairment, freezing of gait, and falls [43, 44]. According to a previous study [9], the demands on the informal caregiver and the estimated cost of informal care are the highest when the patient is in stage IV, as in stage V, it is likely that many patients live in a nursing home.

In only 27 (36%) of the cases, the patient and the informal caregiver had both replied that they were satisfied with their overall care/support. This indicates that there might be a discrepancy between how patients and their informal caregivers, respectively, experience and perceive their situation.

### 4.4. Strengths, Limitations, and Further Perspectives

There are several limitations of this study. It is likely that other or additional factors may be associated with patients' satisfaction with care as well as informal caregivers' satisfaction with support, rather than the ones assessed in the present study. Given the current cross-sectional study design, it is not possible to draw casual conclusions. The symptomatology of late stage PD with increasing cognitive decline and problems in communicating may have affected the quality of the data collected for this study. However, a majority of the participants were able to complete most parts of the study without any apparent compromise in data quality.

The words “care” and “support” were not further defined and could have been interpreted in various ways among the participants. “Overall care” could presumably mean different things, such as home health care or medical care. As a large proportion of the current sample received both home health care and medical care and the proportions of participants were quite evenly distributed between those who were satisfied and those who were not satisfied, the interpretation of the words “care” and “support” would probably not matter for the outcome of this study. Similarly, the word “support” could theoretically mean both emotional/psychological and practical assistance from the health-care system.

A major strength of the present study is that we were able to recruit and assess a relatively large sample of patients in the late and most severe stages of PD, an area where knowledge presently is limited [33]. Future studies on satisfaction with care could further investigate and distinguish between different areas of care instead of focusing on a perception of overall care. Qualitative research methodology may give an insight into which specific areas are important for patient satisfaction with care and informal caregiver satisfaction with support in late stage PD. Availability of health-care and social resources is likely to influence satisfaction with care and support. The results of this study should be replicated in other national contexts, as the current study was carried out in a setting with the Swedish health and social care systems. Moreover, international comparisons would be of value.

## 5. Conclusions

Patient satisfaction with care in late stage PD appears to be negatively associated with depressive symptoms and positively associated with independence in ADL. Satisfaction with somatic health also appears to be of importance for patient satisfaction with care. In addition, late stage patients' informal caregivers' satisfaction with support appears positively associated with patients' HY stage and negatively associated with informal caregivers' QoL.

A multidisciplinary approach to the management of late stage PD patients and their family caregivers should probably have high priority in order to provide an effective symptomatic pharmacological and nonpharmacological PD therapy, to identify and adequately treat motor and NMS (e.g., depressive symptoms), and to offer possibilities for optimal training (e.g., in ADL) in order to optimize management and coping of daily activities. This may benefit not only patients in late stage PD, but also their family caregivers.

## Figures and Tables

**Table 1:** Patients' demographic and clinical data (*n*=107).

	Total cohort	Missing (*n*)
Gender, *n* (%)		—
Men	62 (58%)	
Women	45 (42%)	
Age (years), median (q1–q3)	78 (73–84)	—
Age at onset (years), median (q1–q3)	63 (55–71)	—
PD duration (years), median (q1–q3)	15 (11–19)	—
Dwelling place, *n* (%)		—
Home	67 (63%)	
Nursing home	40 (37%)^*∗*^	
HY stage, median (q1–q3)	4 (4-5)	—
HY stage, *n* (%)		—
IV	79 (74%)	
V	28 (26%)	
Partner, *n* (%)		—
Yes	65 (60%)	
No	42 (39%)	
Professional home health care in home/nursing home, *n* (%)		—
Yes	89 (83%)	
No	18 (17%)	
Professional health-care contact		
MDS and/or PD nurse (past year), *n* (%)		—
Yes	69 (64%)	
No	38 (36%)	
PT and/or OT and/or SLT (past 3 months), *n* (%)		—
Yes	51 (48%)	
No	56 (52%)	
Independence in ADL (Katz ADL index), median (q1–q3)	2 (1–4)	2
Dependent (severe functional impairment; ≤2), *n* (%)	59 (56%)	
Clinical assessments		
Motor function (UPDRS III), median (q1–q3)	40 (29–53)	—
Nonmotor symptoms (NMSS), median (q1–q3)	91 (55–128)	2
Cognitive function (MMSE), median (q1–q3)	22 (18–27)	4
Cognitive impairment (proportion ≤ 23), *n* (%)	60 (58%)	
Depressive symptoms (GDS-30), median (q1–q3)	11 (8–16)	7
Depression (GDS ≥ 10), *n* (%)	62 (62%)	
Health and quality of life related assessments		
Generic health status (EQ-5D), median (q1–q3)	0.19 (0.02–0.53)	5
VAS, median (q1–q3)	50 (30–60)	9
Individual QoL (SEIQoL-Q), median (q1–q3)	55 (43–70)	26
Self-rated health status (NHP), median (q1–q3)		
Emotional reactions	22 (11–56)	12
Sleep	20 (0–60)	8
Energy	67 (33–100)	9
Pain	38 (13–75)	11
Physical mobility	75 (50–88)	9
Social isolation	20 (0–40)	9
NHP index of distress 0–100	33 (17–54)	12
Life satisfaction (LiSat-11), median (q1–q3)		
Life as a whole high/low, *n* (%)	22 (23%)/74 (77%)	11
Life as a whole	4 (3-4)	11
Vocational situation	3 (2–4)	14
Financial situation	5 (4-5)	12
Leisure	3 (2–4)	12
Contacts with friends/acquaintances	4 (3–5)	12
Sexual life	1 (1–3)	12
Self-care management	3 (2-3)	12
Family life	4 (3–5)	12
Partner relationship	4 (1–5)	14
Somatic health	3 (2–4)	12
Psychological health	4 (3–5)	12
Satisfaction with care, *n* (%)		8
Satisfied	58 (59%)	
Not satisfied	41 (41%)	

q1–q3, first and third quartiles; HY, Hoehn and Yahr Staging Scale (score range I–V, higher = worse); MDS, movement disorder specialist contact during the past year; PD-nurse, Parkinson nurse contact during the past year; physiotherapist/occupational therapist/speech and language therapist, contact during the past three months; Katz ADL, Katz index of independence in activities of daily living (score range 0–6, higher = better); UPDRS, Unified PD Rating Scale, part III = motor examination (score range 0–108, higher = worse); NMSS, Nonmotor Symptoms Scale (0–360, higher = worse); MMSE, Mini-Mental State Examination (score range 0–30, higher = better); GDS-30, Geriatric Depression Scale (score range 0––30, higher = worse), depression = scores ≥ 10. EQ-5D-3L, EuroQol 5 Dimensions Index (score range 0-1, higher = better), VAS, visual analogue scale (score range 0–100, higher = better); SEIQoL-Q, Schedule for the Evaluation of Individual Quality of Life (score range 0–100, higher = better); NHP, the Nottingham Health Profile (score range 0–100 in each section, higher = worse); LiSat-11, Life Satisfaction Questionnaire (each item score range 1–6, higher = better); satisfaction with care (study-specific question), satisfied = patients reporting alternative 1 or 2 on the question satisfaction with care (score range 1–5, higher = worse; 1 = very satisfied, 2 = satisfied, 3 = neutral, 4 = unsatisfied, 5 = very unsatisfied), not satisfied = patients reporting alternative 3, 4 or 5 on the question satisfaction with care. ^*∗*^in HY IV 23 (29%) lived in nursing home, in HY V 17 (61%) lived in nursing home.

**Table 2:** Univariate associations with patients' satisfaction with care (*n*=99).

	Satisfied, *n*=58 (59%)	Not satisfied, *n*=41 (41%)	*P* value	Missing (*n*)
Gender, *n* (%)			0.937	—
Men	33 (59%)	23 (41%)		
Women	25 (58%)	18 (42%)		
Age (years), median (q1–q3)	79 (75–84)	78 (73–84)	0.357	—
Age at onset (years), median (q1–q3)	63 (57–70)	63 (53–72)	0.721	—
PD duration (years), median (q1–q3)	15 (12–18)	14 (10–19)	0.644	—
Dwelling place, *n* (%)			0.775	—
Home	37 (60%)	25 (40%)		
Nursing home	21 (57%)	16 (43%)		
HY stage, *n* (%)			0.977	—
IV	44 (59%)	31 (41%)		
V	14 (58%)	10 (42%)		
Partner, *n* (%)			0.631	—
Yes	34 (57%)	26 (43%)		
No	24 (62%)	15 (38%)		
Professional home health care in home/nursing home, *n* (%)			0.773	—
Yes	48 (59%)	33 (41%)		
No	10 (56%)	8 (44%)		
Professional health-care contact				
MDS and/or PD-nurse (past year), *n* (%)			0.773	—
Yes	38 (58%)	28 (42%)		
No	20 (61%)	13 (39%)		
PT and/or OT and/or SLT (past 3 months), *n* (%)			0.270	—
Yes	26 (53%)	23 (47%)		
No	32 (64%)	18 (36%)		
Independence in ADL (Katz ADL index), median (q1–q3)	3 (1–5)	1 (0–4)	**0.044**	1/1
Dependent (severe functional impairment; ≤ 2), *n* (%)	25 (49%)	26 (51%)	**0.042**	
Clinical assessments				
Motor function (UPDRS III), median (q1–q3)	40 (29–51)	37 (27–52)	0.901	—
Nonmotor symptoms (NMSS), median (q1–q3)	85 (52–121)	92 (61–133)	0.414	—
Cognitive function (MMSE), median (q1–q3)	23 (18–26)	24 (19.5–28)	0.282	2/-
Depressive symptoms (GDS–30), median (q1–q3)	10 (6–13)	14 (10–19)	**0.005**	2/-
Depression (GDS ≥ 10), *n* (%)	29 (48%)	31 (52%)	**0.019**	
Health and quality of life related assessments				
Generic health status (EQ-5D), median (q1–q3)	0.23 (0.07–0.62)	0.15 (−0.02–0.52)	0.062	—
VAS, median (q1–q3)	50 (35–67)	45 (30–60)	0.176	3/-
Individual QoL (SEIQoL-Q), median (q1–q3)	61 (46–74)	51 (39–62)	**0.036**	12/7
Self-rated health status (NHP), *n* (%)				
Emotional reactions: better/worse	39 (59%)/13 (52%)	27 (41%)/12 (48%)	0.542	6/2
Sleep: better/worse	41 (59%)/15 (58%)	28 (41%)/11 (42%)	0.879	2/2
Energy: better/worse	23 (72%)/32 (52%)	9 (28%)/30 (48%)	0.062	3/2
Pain: better/worse	36 (62%)/19 (56%)	22 (38%)/15 (44%)	0.559	3/4
Physical mobility: better/worse	16 (62%)/40 (59%)	10 (38%)/28 (41%)	0.810	2/3
Social isolation: better/worse	46 (62%)/9 (45%)	28 (38%)/11 (55%)	0.171	3/2
NHP index of distress 0–100: better/worse	40 (61%)/12 (48%)	26 (39%)/13 (52%)	0.337	6/2
Life satisfaction (LiSat-11), *n* (%)				
Life as a whole: high/low	16 (76%)/38 (52%)	5 (24%)/35 (48%)	0.055	4/1
Vocational situation: high/low	6 (60%)/46 (56%)	4 (40%)/36 (44%)	0.814	7/1
Financial situation: high/low	35 (65%)/18 (46%)	19 (35%)/21 (54%)	0.075	5/1
Leisure: high/low	10 (63%)/43 (56%)	6 (38%)/34 (44%)	0.625	5/1
Contacts with friends/acquaintances: high/low	25 (63%)/28 (53%)	15 (38%)/25 (47%)	0.352	5/1
Sexual life: high/low	6 (67%)/47 (56%)	3 (33%)/37 (44%)	0.540	5/1
Self-care management: high/low	6 (86%)/47 (55%)	1 (14%)/39 (45%)	0.145	5/1
Family life: high/low	29 (64%)/24 (50%)	16 (36%)/24 (50%)	0.161	5/1
Partner relationship: high/low	25 (64%)/28 (53%)	14 (36%)/25 (47%)	0.282	5/2
Somatic health: high/low	9 (90%)/44 (53%)	1 (10%)/39 (47%)	0.054	5/1
Psychological health: high/low	22 (65%)/31 (53%)	12 (35%)/28 (47%)	0.256	5/1

Satisfaction with care (study-specific question), satisfied = patients reporting alternative 1 or 2 on the question satisfaction with care (score range 1–5, higher = worse; 1 = very satisfied, 2 = satisfied, 3 = neutral, 4 = unsatisfied, 5 = very unsatisfied), not satisfied = patients reporting alternative 3, 4, or 5 on the question satisfaction with care. q1–q3, first and third quartiles; HY, Hoehn and Yahr Staging Scale (score range I–V, higher = worse); MDS, movement disorder specialist contact during the past year; PD-nurse, Parkinson nurse contact during the past year; physiotherapist/occupational therapist/speech and language therapist, contact during the past three months; Katz ADL, Katz index of independence in activities of daily living (score range 0–6, higher = better); UPDRS, Unified PD Rating Scale, part III = motor examination (score range 0–108, higher = worse); NMSS, Nonmotor Symptoms Scale (0–360, higher = worse); MMSE, Mini-Mental State Examination (score range 0–30, higher = better); GDS-30, Geriatric Depression Scale (score range 0–30, higher = worse), depression = scores ≥ 10. EQ-5D-3L, EuroQol 5 Dimensions Index (score range 0–1, higher = better); VAS, visual analogue scale (score range 0–100, higher = better); SEIQoL-Q, Schedule for the Evaluation of Individual Quality of Life (score range 0–100, higher = better); NHP, the Nottingham Health Profile (score range 0–100 in each section, higher = worse; dichotomized as better = 0, worse = 1); LiSat-11, Life Satisfaction Questionnaire (each item score range 1–6, higher = better; dichotomized as not satisfied = 0, satisfied = 1). *P* values based on simple logistic regression analyses. Bold *P* values statistically significant at *P* < 0.05.

**Table 3:** Univariate associations with informal caregivers' satisfaction with support (*n*=76^*∗*^).

	Satisfied, *n*=45 (59%)	Not satisfied, *n*=31 (41%)	*P* value	Missing (*n*)
Patient demographics and clinical data				
Patient gender, *n* (%)			0.839	—
Men	28 (58%)	20 (42%)		
Women	17 (61%)	11 (39%)		
Patient age, median (q1–q3)	80 (76–85)	75 (71–80)	**0.009**	—
Patient dwelling place, *n* (%)			0.054	—
Home	25 (51%)	24 (49%)		
Nursing home	20 (74%)^*∗∗*^	7 (26%)		
HY stage, *n* (%)			**0.004**	—
IV	27 (48%)	29 (52%)^*∗∗∗*^		
V	18 (90%)^*∗∗∗∗*^	2 (10%)		
Patient has professional home health care in home/nursing home, *n* (%)			0.251	—
Yes	37 (63%)	22 (37%)		
No	8 (47%)	9 (53%)		
Professional health-care contact				
MDS and/or PD-nurse (past year), *n* (%)			0.086	—
Yes	23 (51%)	22 (49%)		
No	22 (71%)	9 (29%)		
PT and/or OT and/or SLT (past 3 months), *n* (%)			0.828	—
Yes	20 (61%)	13 (39%)		—
No	25 (58%)	18 (42%)		—
Patient independence in ADL (Katz ADL index), median (q1–q3)	2 (1–5)	1.5 (0.75–4)	0.449	1/1
Dependent (severe functional impairment; ≤2), *n* (%)	23 (56%)	18 (44%)	0.512	
Patient clinical assessments				
Motor function (UPDRS III), median (q1–q3)	41 (33–57)	33 (26–42)	**0.015**	—
Nonmotor symptoms (NMSS), median (q1–q3)	100 (49–138)	105 (61–139)	0.831	2/-
Cognitive function (MMSE), median (q1–q3)	21 (18–25)	22 (19–26)	0.223	4/-
Depressive symptoms (GDS–30), median (q1–q3)	11 (8–15)	12 (8–19)	0.328	6/1
Depression (GDS ≥ 10), *n* (%)	25 (56%)	20 (44%)	0.825	
Patient health and quality of life related assessments				
Generic health status (EQ–5D), median (q1–q3)	0.19 (0.08–0.52)	0.39 (0.07–0.62)	0.118	3/1
VAS, median (q1–q3)	50 (40–60)	50 (30–60)	0.743	6/2
Individual QoL (SEIQoL), median (q1–q3)	0.57 (0.41–0.75)	0.55 (0.51–0.62)	0.623	15/6
Life satisfaction (LiSat-11), median (q1–q3)				
Life as a whole, *n* (%)			0.589	7/1
Satisfied	17 (55%)	14 (45%)		
Not satisfied	15 (52%)	14 (48%)		
Patient satisfaction with care, *n* (%)			0.142	5/1
Satisfied	27 (64%)	15 (36%)		
Not satisfied	13 (46%)	15 (54%)		
Caregiver quality of life				
ACQLI total score, median (q1–q3)	7 (3–12)	13 (7–19)	**0.036**	2/1

Satisfaction with support (study-specific question), satisfied = informal caregivers reporting alternative 1 or 2 on the question satisfaction with care (score range 1–5, higher = worse; 1 = very satisfied, 2 = satisfied, 3 = neutral, 4 = unsatisfied, 5 = very unsatisfied), not satisfied = informal caregivers reporting alternative 3, 4, or 5 on the question satisfaction with care. q1–q3, first and third quartiles; HY, Hoehn and Yahr Staging Scale (score range I–V, higher = worse); MDS, movement disorder specialist contact during the past year; PD-nurse, Parkinson nurse contact during the past year; physiotherapist/occupational therapist/speech and language therapist, contact during the past three months; Katz ADL, Katz index of independence in activities of daily living (score range 0–6, higher = better); UPDRS, Unified PD Rating Scale, part III = motor examination (score range 0–108, higher = worse); NMSS, Nonmotor Symptoms Scale (0–360, higher = worse); MMSE, Mini-Mental State Examination (score range 0–30, higher = better); GDS-30, Geriatric Depression Scale (score range 0–30, higher = worse), depression = scores ≥ 10. EQ-5D-3L, EuroQol 5 Dimensions Index (score range 0–1, higher = better); VAS, visual analogue scale (score range 0–100, higher = better); SEIQoL-Q, Schedule for the Evaluation of Individual Quality of Life index (score range 0–100, higher = better); LiSat-11, Life Satisfaction Questionnaire (item 1, score range 1–6; dichotomized as not satisfied = 0, satisfied = 1) ACQLI, Alzheimer's Carer's Quality of Life Inventory (score range 0–30, higher = worse). ^*∗*^30 missing (primarily due to nonapplicable). ^*∗∗*^HY IV *n*=10, HY V *n*=10; ^*∗∗∗*^6 (21%) of the patients living in nursing home; ^*∗∗∗∗*^10 (56%) of the patients living in nursing home. *P* values based on simple logistic regression analyses. Bold *P* values statistically significant at *P* < 0.05.

**Table 4:** Multivariable logistic regression model of associations with patient satisfaction with care (not satisfied = 0; satisfied = 1), *n*=90.

Independent variables^1^	OR (95% CI)	Wald	*P* value
Depressive symptoms (GDS-30)	0.91 (0.84–0.98)	5.87	**0.015**
Independence in ADL (Katz index)	1.31 (1.04–1.66)	5.06	**0.025**
Satisfaction with somatic health (LiSat-11)	8.01 (0.82–78.74)	3.18	0.074

OR, odds ratio; CI, confidence interval; ^1^independent variables entered in the multivariable logistic regression model (backward method): Housing, partner, independence in ADL (Katz index), depressive symptoms (GDS-30), generic health status (EQ-5D), energy (self-rated health status, NHP; dichotomized as better = 0, worse = 1), social isolation (self-rated health status, NHP; dichotomized as better = 0, worse = 1); satisfaction with life as a whole (LiSat-11; dichotomized as not satisfied = 0, satisfied = 1); satisfaction with financial situation (LiSat-11; dichotomized as not satisfied = 0, satisfied = 1); satisfaction with self-care management (LiSat-11; dichotomized as not satisfied = 0, satisfied = 1); satisfaction with family life (LiSat-11; dichotomized as not satisfied = 0, satisfied = 1); satisfaction with somatic health (LiSat-11; dichotomized as not satisfied = 0, satisfied = 1). Nagelkerke's pseudo *R*^2^ = 0.226; Hosmer and Lemeshow test: *P*=0.450. Bold *P* values statistically significant at *P* < 0.05.

**Table 5:** Multivariable logistic regression model of associations with informal caregivers' satisfaction with support (not satisfied = 0; satisfied = 1), *n*=68.

Independent variables^1^	OR (95% CI)	Wald	*P* value
Patient HY stage	12.15 (2.14–68.90)	7.95	**0.005**
Caregiver QoL (ACQLI)	0.90 (0.83–0.98)	6.32	**0.012**

OR, odds ratio; CI, confidence interval; ^1^independent variables entered in the multivariable logistic regression model (backward method): age of the patient; housing of the patient; HY stage of the patient; professional home health care; MDS/PD-nurse contact past year; motor function of the patient (UPDRS III); generic health status of the patient (EQ-5D); patient satisfaction with care; caregiver QoL (ACQLI). Nagelkerke's pseudo *R*^2^ = 0.313; Hosmer and Lemeshow test: *P*=0.460. Bold *P* values statistically significant at *P* < 0.05.

**Table 6:** Informal caregivers' satisfaction with support, when the patient lives at home (*n* = 49).

	Satisfied, *n*=25 (51%)	Not satisfied, *n*=24 (49%)	*P* value	Missing (*n*)
HY stage, *n* (%)			**0.032**	—
IV	17 (43%)	23 (58%)		
V	8 (89%)	1 (11%)		
*Patient clinical assessments*				
Motor function (UPDRS III), median (q1–q3)	37 (31–54)	32 (26–42)	0.092	—
Nonmotor symptoms (NMSS), median (q1–q3)	89 (47–133)	93 (60–118)	0.621	1/-
Cognitive function (MMSE), median (q1–q3)	23 (21–28)	23 (19–27)	0.521	3/-
Depressive symptoms (GDS-30), median (q1–q3)	10 (8–18)	12 (8–20)	0.686	3/1
Depression (GDS ≥10), *n* (%)	13 (48%)	14 (52%)	0.903	

Satisfaction with support (study-specific question), satisfied = patients reporting alternative 1 or 2 on the question satisfaction with care (score range 1–5, higher = worse; 1 = very satisfied, 2 = satisfied, 3 = neutral, 4 = unsatisfied, 5 = very unsatisfied), not satisfied = patients reporting alternative 3, 4, or 5 on the question satisfaction with care. q1–q3, first and third quartiles; HY, Hoehn and Yahr Staging Scale (score range I–V, higher = worse); UPDRS, Unified PD Rating Scale, part III = motor examination (score range 0–108, higher = worse); NMSS, Nonmotor Symptoms Scale (0–360, higher = worse); MMSE, Mini-Mental State Examination (score range 0–30, higher = better); GDS-30, Geriatric Depression Scale (score range 0–30, higher = worse), depression = scores ≥ 10. Bold *P* values statistically significant at *P* < 0.05.

## Data Availability

The data used to support the findings of this study are available from the corresponding author upon request.

## References

[1] Hoehn M. M., Yahr M. D. (1967). Parkinsonism: onset, progression, and mortality. *Neurology*.

[2] Rosqvist K., Horne M., Hagell P., Iwarsson S., Nilsson M. H., Odin P. (2018). Levodopa effect and motor function in late stage Parkinson’s disease. *Journal of Parkinson’s Disease*.

[3] Rosqvist K., Odin P., Hagell P., Iwarsson S., Nilsson M. H., Storch A. (2018). Dopaminergic effect on non-motor symptoms in late stage Parkinson’s disease. *Journal of Parkinson’s Disease*.

[4] Weerkamp N. J., Tissingh G., Poels P. J. E. (2013). Nonmotor symptoms in nursing home residents with Parkinson’s disease: prevalence and effect on quality of life. *Journal of the American Geriatrics Society*.

[5] Coelho M., Ferreira J. J. (2012). Late-stage Parkinson disease. *Nature Reviews Neurology*.

[6] Enders D., Balzer-Geldsetzer M., Riedel O. (2017). Prevalence, duration and severity of Parkinson’s disease in Germany: a combined meta-analysis from literature data and outpatient samples. *European Neurology*.

[7] Bach J.-P., Ziegler U., Deuschl G., Dodel R., Doblhammer-Reiter G. (2011). Projected numbers of people with movement disorders in the years 2030 and 2050. *Movement Disorders*.

[8] Dorsey E. R., Constantinescu R., Thompson J. P. (2007). Projected number of people with Parkinson disease in the most populous nations, 2005 through 2030. *Neurology*.

[9] Mosley P. E., Moodie R., Dissanayaka N. (2017). Caregiver burden in Parkinson disease: a critical review of recent literature. *Journal of Geriatric Psychiatry and Neurology*.

[10] Page T. E., Farina N., Brown A. (2017). Instruments measuring the disease-specific quality of life of family carers of people with neurodegenerative diseases: a systematic review. *BMJ Open*.

[11] Schrag A., Hovris A., Morley D., Quinn N., Jahanshahi M. (2006). Caregiver-burden in Parkinson’s disease is closely associated with psychiatric symptoms, falls, and disability. *Parkinsonism & Related Disorders*.

[12] Schrag A., Khan K., Hotham S., Merritt R., Rascol O., Graham L. (2018). Experience of care for Parkinson’s disease in european countries: a survey by the european Parkinson’s disease association. *European Journal of Neurology*.

[13] Dorsey E. R., Voss T. S., Shprecher D. R. (2010). A U.S. survey of patients with Parkinson’s disease: satisfaction with medical care and support groups. *Movement Disorders*.

[14] Schwab R., England A., Gillingham F. J., Donaldson M. C. Projection technique for evaluating surgery in Parkinson’s disease.

[15] Balzer-Geldsetzer M., Ferreira J., Odin P. (2018). Study protocol: Care of Late-Stage Parkinsonism (CLaSP): a longitudinal cohort study. *BMC Neurology*.

[16] Katz S., Ford A. B., Moskowitz R. W., Jackson B. A., Jaffe M. W. (1963). Studies of illness in the aged. *JAMA*.

[17] Fahn S., Elton R. L., Members of the UPDRS Development Committee, Fahn S., Marsden C., Calne D., Goldstein M. (1987). Unified Parkinson’s disease rating scale. *Recent Developments in Parkinson’s Disease*.

[18] Chaudhuri K. R., Martinez-Martin P., Brown R. G. (2007). The metric properties of a novel non-motor symptoms scale for Parkinson’s disease: results from an international pilot study. *Movement Disorders*.

[19] Folstein M. F., Folstein S. E., McHugh P. R. (1975). Mini-mental state. *Journal of Psychiatric Research*.

[20] Dick J. P., Guiloff R. J., Stewart A. (1984). Mini-mental state examination in neurological patients. *Journal of Neurology, Neurosurgery & Psychiatry*.

[21] Yesavage J. A., Brink T. L., Rose T. L. (1982). Development and validation of a geriatric depression screening scale: a preliminary report. *Journal of Psychiatric Research*.

[22] McDonald W. M., Holtzheimer P. E., Haber M., Vitek J. L., McWhorter K., Delong M. (2006). Validity of the 30-item geriatric depression scale in patients with Parkinson’s disease. *Movement Disorders*.

[23] Schrag A., Selai C., Jahanshahi M., Quinn N. P. (2000). The EQ-5D—a generic quality of life measure—is a useful instrument to measure quality of life in patients with Parkinson’s disease. *Journal of Neurology, Neurosurgery & Psychiatry*.

[24] EQ-5D-3L https://euroqol.org/eq-5d-instruments/eq-5d-3l-about/.

[25] Becker G., Merk C. S., Meffert C., Momm F. (2014). Measuring individual quality of life in patients receiving radiation therapy: the SEIQoL-Questionnaire. *Quality of Life Research*.

[26] Hunt S. M., McEwen J., McKenna S. P. (1985). Measuring health status: a new tool for clinicians and epidemiologists. *Journal of the Royal College of General Practitioners*.

[27] Wiklund I., Romanus B., Hunt S. M. (1988). Self-assessed disability in patients with arthrosis of the hip joint: reliability of the Swedish version of the Nottingham health profile. *International Disability Studies*.

[28] Hagell P., Whalley D., McKenna S. P., Lindvall O. (2003). Health status measurement in Parkinson’s disease: validity of the PDQ-39 and Nottingham health profile. *Movement Disorders*.

[29] Fugl-Meyer A. R., Melin R., Fugl-Meyer K. S. (2002). Life satisfaction in 18- to 64-year-old Swedes: in relation to gender, age, partner and immigrant status. *Journal of Rehabilitation Medicine*.

[30] Gustafsson H., Nordström P., Stråhle S., Nordström A. (2015). Parkinson’s disease: a population-based investigation of life satisfaction and employment. *Journal of Rehabilitation Medicine*.

[31] Rosqvist K., Hagell P., Odin P., Ekström H., Iwarsson S., Nilsson M. H. (2017). Factors associated with life satisfaction in Parkinson’s disease. *Acta Neurologica Scandinavica*.

[32] Hagell P., Smith S. (2013). A psychometric comparison of two carer quality of life questionnaires in Huntington’s disease: implications for neurodegenerative disorders. *Journal of Huntington’s Disease*.

[33] Coelho M., Marti M. J., Tolosa E. (2010). Late-stage Parkinson’s disease: the barcelona and lisbon cohort. *Journal of Neurology*.

[34] Fabbri M., Coelho M., Guedes L. C. (2017). Response of non-motor symptoms to levodopa in late-stage Parkinson’s disease: results of a levodopa challenge test. *Parkinsonism & Related Disorders*.

[35] Weintraub D., Moberg P. J., Duda J. E., Katz I. R., Stern M. B. (2004). Effect of psychiatric and other nonmotor symptoms on disability in Parkinson’s disease. *Journal of the American Geriatrics Society*.

[36] Soh S.-E., Morris M. E., McGinley J. L. (2011). Determinants of health-related quality of life in Parkinson’s disease: a systematic review. *Parkinsonism & Related Disorders*.

[37] Troeung L., Egan S. J., Gasson N. (2013). A meta-analysis of randomised placebo-controlled treatment trials for depression and anxiety in Parkinson’s disease. *PLoS One*.

[38] Reynolds G. O., Otto M. W., Ellis T. D., Cronin-Golomb A. (2016). The therapeutic potential of exercise to improve mood, cognition, and sleep in Parkinson’s disease. *Movement Disorders*.

[39] Mueller C., Rajkumar A. P., Wan Y. M. (2018). Assessment and management of neuropsychiatric symptoms in Parkinson’s disease. *CNS Drugs*.

[40] Classen J., Koschel J., Oehlwein C. (2017). Nonmotor fluctuations: phenotypes, pathophysiology, management, and open issues. *Journal of Neural Transmission*.

[41] Varanese S., Birnbaum Z., Rossi R., Di Rocco A. (2011). Treatment of advanced Parkinson’s disease. *Parkinson’s Disease*.

[42] The National Swedish Guidelines for PD https://www.socialstyrelsen.se/SiteCollectionDocuments/2016-12-1-Kunskapsunderlag-PD.pdf.

[43] Bloem B. R., Hausdorff J. M., Visser J. E., Giladi N. (2004). Falls and freezing of gait in Parkinson’s disease: a review of two interconnected, episodic phenomena. *Movement Disorders*.

[44] Okuma Y. (2014). Freezing of gait and falls in Parkinson’s disease. *Journal of Parkinson’s Disease*.

